# Sheep Face Recognition Model Based on Deep Learning and Bilinear Feature Fusion

**DOI:** 10.3390/ani13121957

**Published:** 2023-06-11

**Authors:** Zhuang Wan, Fang Tian, Cheng Zhang

**Affiliations:** 1 College of Informatics, Huazhong Agricultural University, Wuhan 430070, China; 2 Key Laboratory of Smart Animal Farming Technology, Ministry of Agriculture and Rural Affairs, Huazhong Agricultural University, Wuhan 430070, China; 3 Hubei Engineering Technology Research Center of Agricultural Big Data, Huazhong Agricultural University, Wuhan 430070, China

**Keywords:** sheep face recognition, deep learning, feature fusion, RepVGG

## Abstract

**Simple Summary:**

Identifying individual sheep accurately is crucial for establishing precise animal husbandry. In the process of identifying sheep by their faces, changes in sheep face poses and different camera angles can affect the identification accuracy. In this study, we construct a new sheep face recognition model. Sheep face data with different poses and angles are used as input in a bilinear feature extraction network, which extracts the important features of sheep faces separately. Then, a feature fusion method is used to fuse the features extracted by the bilinear network for sheep face recognition. Our experimental results demonstrate that the recognition accuracy of the algorithm is 99.43%, achieving the individual recognition of sheep in complex environments while reducing the influence of pose and angle on recognition.

**Abstract:**

A key prerequisite for the establishment of digitalized sheep farms and precision animal husbandry is the accurate identification of each sheep’s identity. Due to the uncertainty in recognizing sheep faces, the differences in sheep posture and shooting angle in the recognition process have an impact on the recognition accuracy. In this study, we propose a deep learning model based on the RepVGG algorithm and bilinear feature extraction and fusion for the recognition of sheep faces. The model training and testing datasets consist of photos of sheep faces at different distances and angles. We first design a feature extraction channel with an attention mechanism and RepVGG blocks. The RepVGG block reparameterization mechanism is used to achieve lossless compression of the model, thus improving its recognition efficiency. Second, two feature extraction channels are used to form a bilinear feature extraction network, which extracts important features for different poses and angles of the sheep face. Finally, features at the same scale from different images are fused to enhance the feature information, improving the recognition ability and robustness of the network. The test results demonstrate that the proposed model can effectively reduce the effect of sheep face pose on the recognition accuracy, with recognition rates reaching 95.95%, 97.64%, and 99.43% for the sheep side-, front-, and full-face datasets, respectively, outperforming several state-of-the-art sheep face recognition models.

## 1. Introduction

With modern technological advances, the trend of transitioning from small-scale and free-range systems to intensive and smart systems for sheep farming is accelerating [1]. The precise identification of individual sheep is a fundamental prerequisite for smart farming, playing a crucial role in individual sheep growth records, breeding management, health status, and behavior analysis [2]. Livestock farms typically use visual inspection [3] or sensors [4] such as accelerometers, rumen pH sensors, and thermometers to assess the physical condition of livestock. When sick livestock are found, they need to be identified by the breeder at close range and registered for subsequent treatment and handling. This is an inefficient method of identification, which also puts the breeder at risk of contracting zoonotic diseases [5].

Physical identification techniques, such as dye marking, hot and cold branding [6], ear clipping and marking [7], ear tagging, and radio frequency identification (RFID) [8], can be employed for livestock identification. According to the research situations of several sheep farms in Gansu, China, ear tag identification methods such as breeding ear tags, electronic ear tags, and embedded chips are most used in medium- and large-sized sheep farms. However, ear tags have problems such as shedding and ear tag number wear [9]. Some sheep need to wear more than one ear tag, which may produce stress, posing problems for sheep welfare and breeding [10]. Biometric and computer vision technologies [11], such as iris recognition [12], retinal recognition [13], and facial recognition [14], are non-contact identification technologies that can address the limitations of ear tags. Iris and retinal recognition technologies, although capable of achieving higher accuracy rates, are not easily applicable in larger sheep farms due to the expensive equipment and rigorous collection requirements they demand. In contrast, facial recognition technology can be implemented with cheaper cameras and boasts a wealth of facial data. Through the use of computer vision technology, individual sheep can be accurately identified in real time without posing an economic burden on farms.

There has been a recent surge in scholars utilizing computer vision techniques to recognize animal faces. For example, Corkey et al. used an independent component analysis technique with a cosine distance classifier to achieve the facial recognition of 50 sheep with a recognition accuracy of 95–96.3% [15]; however, only 2–3 images were used for each sheep and some distracting factors, such as lighting, were not excluded. In 2005, Kim et al. learned the faces of 12 Japanese black cattle using an associative memory neural network method and verified the recognition performance on transformed images. The results indicated that the recognition effect was good, but the model cannot address real-time data and, thus, could not yet be applied in cattle farms [16]. Cai et al. improved the local binary pattern (LBP) bull face recognition method on the basis of facial recognition, which could recognize gray bull face pictures after calibration using sparse and low-rank decomposition [17]. Kumar et al. extracted features through an LBP and accelerated the robust feature technology, and then used weighted and rule fusion technology to combine and fuse the features for cow face recognition, where the recognition accuracy rate reached 92.5% [18]. Naoki et al. achieved the facial recognition of 16 pigs using an eigenspace method and principal component analysis (PCA), yielding an accuracy of 97.9%, but the similarity of the dataset and data augmentation were not considered, and the method was not robust [19].

Convolutional neural networks (CNNs) are some of the most critical networks in the field of deep learning, which can extract important features from data [20]. The advancement of CNN technology in recent years has prompted a growing number of scholars to apply this technology in the field of livestock facial recognition. Hansen et al. tested 10 pigs using three recognition techniques [21]. The results indicated that their self-built convolutional neural network model performed the best in recognizing 1553 pig face images collected from the top view with 96.7% accuracy. However, as the captured camera was fixed to collect the front-face images of pigs during drinking, images from different angles were not verified. Wang et al. applied stochastic gradient descent (SGD), adaptive moment estimation (Adam), and root mean square prop (RMSprop) optimizers to the Lenet-5 model to recognize pig faces, where the SGD optimizer presented the best recognition accuracy of 97.6% [22]. Marsot et al. proposed a new framework to first detect the eyes and faces of pigs using the modified Haar cascade method and a shallow CNN to obtain high-quality pictures of pig faces, which was followed by a deep convolutional neural network to achieve the facial recognition of 10 pigs with 83% recognition accuracy [23]. Yao et al. combined the detection-type Faster R-CNN with the PnasNet5 model to achieve recognition of cattle images [24]. Wang et al. used migration learning to pretrain the initial weights using the VGGFace dataset and update the weights on a bull face dataset. The resulting accuracy of the model on the bull face dataset was 93% [25]. Salama et al. used Bayesian optimization to update the parameters of the CNN to achieve 98% accuracy in the recognition of sheep faces [26]. Xue et al. proposed a sheep face recognition model that first aligns sheep faces to the horizontal direction, then extracts features using a CNN, and finally processes the features into Euclidean space vectors to recognize sheep faces [27].

The recognition of sheep faces is similar to that for humans [15], and the utilization of deep learning technology is considered the primary direction for future sheep facial recognition research [28]. Sheep faces are influenced by factors such as hair, texture, gesture changes, perspective, and complex backgrounds, which can make recognition challenging. Moreover, capturing sheep face images is complicated due to the uncontrollable nature of the animal’s movements during acquisition, resulting in unbalanced data from various angles with significant differences. Most current studies focus on frontal face data, with few examining the sides of faces or faces from different angles. To solve this problem, we construct three sheep face datasets and propose a sheep face recognition model based on bilinear feature extraction. The proposed model employs a backbone network with two feature extraction branches, each composed of an SA spatial channel-mixing attention mechanism [29] and RepVGG blocks. The SA block can improve the feature extraction ability of the network, while the reparameterization property of the RepVGG block can assist the network in achieving lossless compression, thus reducing model recognition time and improving detection efficiency. The two feature extraction channels form a bilinear model, which can extract and fuse important features of sheep faces with different postures and angles for recognition, solving the problem of missing partial features of a single sheep face due to differences in posture.

## 2. Materials and Methods

### 2.1. Data Collection and Processing

**Figure 1:**
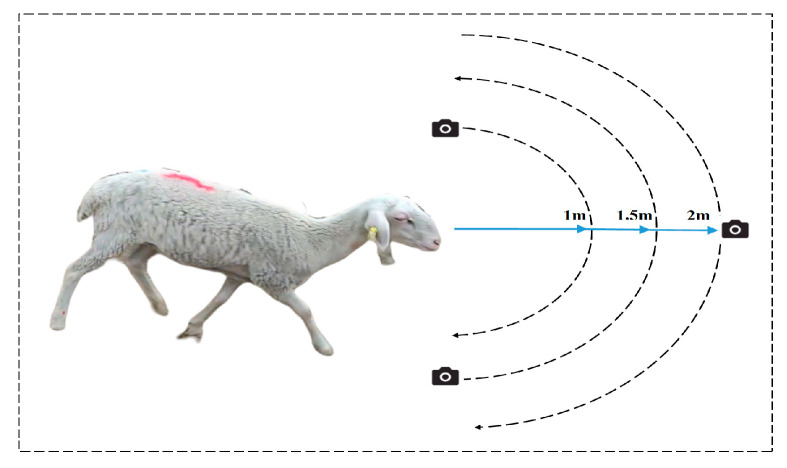
Sheep face data collection process.

The data collected for the experiment were obtained between 8:00 a.m. and 11:30 a.m., as well as between 2:00 p.m. and 5:00 p.m., each day from 25 August 2020 to 31 August 2020. The research subjects were Hu sheep, with ages ranging from six months to two years. The number of Hu sheep utilized was 46, and the data were collected in Jinchang, Gansu Province, China. The data were obtained by recording videos (at 30 frames per second) using a Huawei Mate 30 phone camera. The data acquisition process is depicted in Figure 1. The data acquisition process was guided by three principles. First, to account for the multiscale problem, sheep face photos were obtained from three different distances. Second, to consider the different angles and postures, the sheep face was fixed at an angle and the handheld camera was used to capture images surrounding the sheep face. Third, in consideration of lighting differences, the images were captured under varying lighting conditions, including shadowed, occluded, and indoor–outdoor settings.

**Figure 2:**
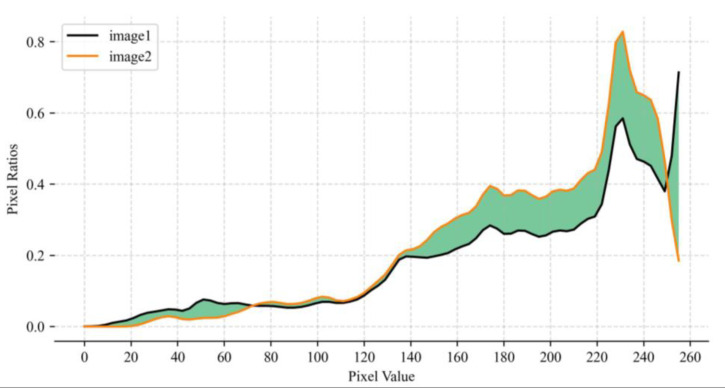
Two processed images. The *x*-axis refers to the pixel values (between 0 and 255) in the image, while the *y*-axis refers to the proportion of each pixel value.

First, the sheep face video was processed into images, and the images without sheep faces or incomplete facial information were eliminated. Then, to remove the interference of the background, the YOLOv5s object detection algorithm [30] was used to detect the sheep face. The detected sheep face was cropped from the original image and the face image was divided into a front image of the sheep and a side image of the sheep based on whether two eyes are visible. Second, considering the high similarity between consecutive frames of sheep face images, we converted the sheep face images into histograms and then normalized them, as shown in Figure 2.

Equation (1) was used to calculate the similarity *S* between two sheep face images, and images with an *S* greater than 0.8 (i.e., the similarity threshold) were eliminated. In Equation (1), $g_{i}$ and $s_{i}$ represent the histogram values of the two pictures in the *i*th dimension.

(1) $$S=\frac{1}{N}\sum_{i=1}^{N}\left(1-\frac{\left|g_{i}-s_{i}\right|}{Max(g_{i},s_{i})}\right)$$

**Figure 3:**
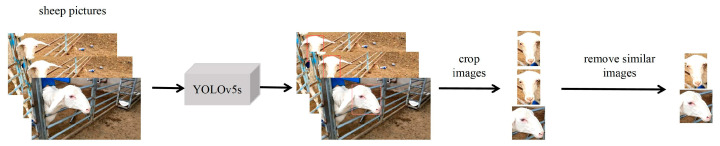
Data processing process.

After eliminating similar images, we constructed a sheep front-face dataset, a sheep side-face dataset, and a sheep full-face dataset based on 40 randomly selected sheep to fit various experimental scenarios. These datasets consisted of 12,538 sheep face images in total, including 6269 front-face images and 6269 side-face images. The data preprocessing process is shown in Figure 3.

**Figure 4:**
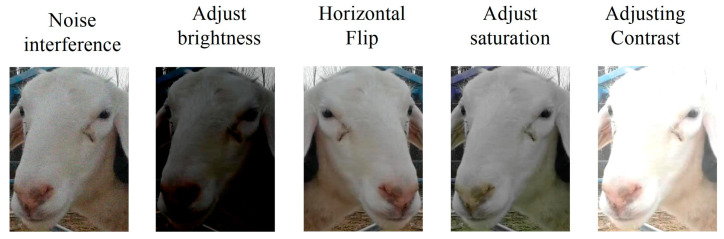
Sample sheep face image data enhancement results.

In order to improve the robustness of the model, five methods were used to augment the sheep face dataset during the training process, including noise interference, random adjustment of brightness, horizontal flipping, random adjustment of saturation, and random adjustment of contrast. The data augmentation effects are depicted in Figure 4.

### 2.2. Bilinear Feature Extraction and Fusion Model

**Figure 5:**
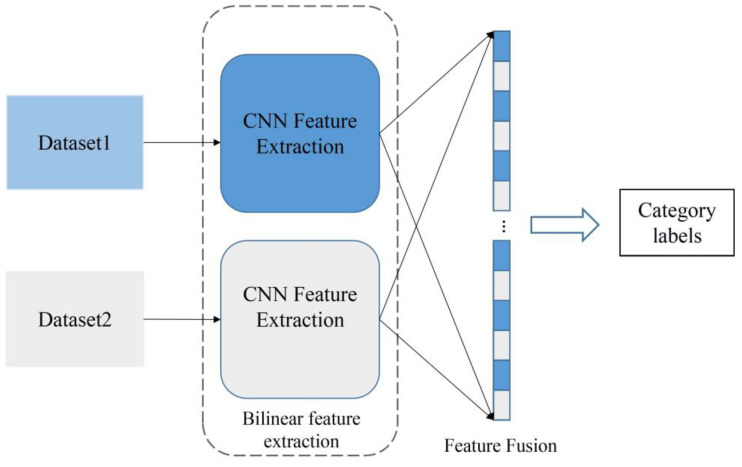
Bilinear feature extraction structure.

Traditional CNN models are basically single-channel structures, such as Alexnet, VGG, Resnet, and so on. A single-branch CNN takes only one input at a time, and the information is processed by the convolutional, activation function and the pooling layers in the CNN to obtain a single feature. In recent years, multichannel neural network models have also been proposed and utilized [31,32]. Unlike single-channel neural networks, multichannel neural network models can have multiple information inputs. As the fusion of multiple features can complement each other with relevant information and effectively improve the recognition accuracy, we used a bilinear feature extraction channel to construct the proposed model. The bilinear feature extraction model consists of two input branches, through which the features of different angles of the image are extracted separately for fusion. The structure is shown in Figure 5.

### 2.3. RepVGG Block

**Figure 6:**
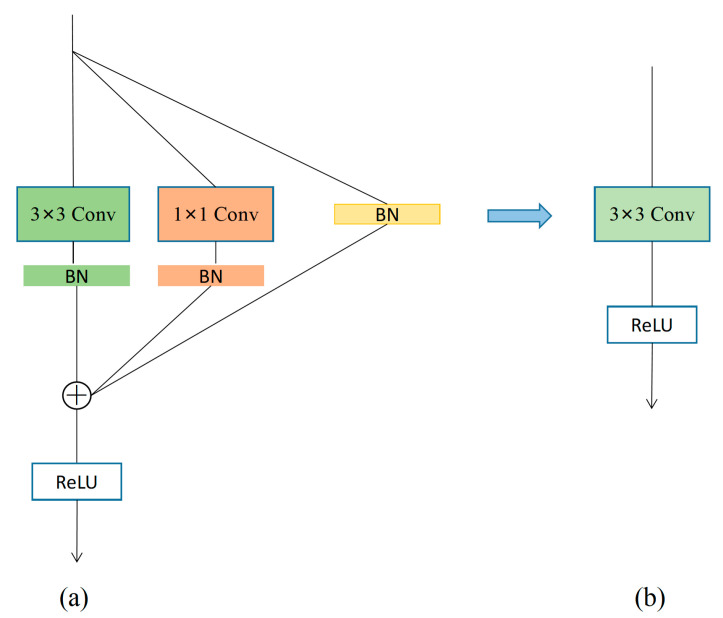
Model structure of RepVGG module: (**a**) training model structure, where the convolution part contains 1 × 1 convolution, 3 × 3 convolution, and batch normalization (BN) layers; and (**b**) inference model structure, where the convolution part uses only a 3 × 3 convolution layer.

RepVGG [33] is an improved backbone network based on the VGG network [34], with the whole model having a simple structure. RepVGG uses a multibranch model structure for training and a single-branch model structure for inference, where the conversion between these two structures is carried out by structural reparameterization. The training structure consists of 1 × 1 convolution, 3 × 3 convolution, and residual branching; a partial representation of the training state model of the RepVGG block is shown in Figure 6a. Meanwhile, the structure of the inference state model of the RepVGG block consists of 3 × 3 convolution as well as ReLU, as shown in Figure 6b.

In our model, we used a RepVGG block instead of traditional convolution in order to improve model accuracy and speed up the inference time. The RepVGG architecture adopted a simplified structure based on VGG, using only 1 × 1 convolutions, 3 × 3 convolutions, and residual branches during training. The 3 × 3 convolutions are computationally efficient and have high computation density, while the residual branches help the network to increase its depth and extract richer features. The RepVGG blocks have the characteristic of structural reparameterization, through which the learned model parameters can be merged and combined, enabling lossless compression of the model without compromising accuracy, thereby speeding up the inference time and improving recognition speed.

When the input features and output features have the same height, width, and number of channels, the RepVGG block can be converted from the training state to the inference state, with the change in model parameters during the conversion being expressed in Equation (2):

(2) $$\begin{array}{ll} M_{2} & =\;BN(M_{1}*W^{\left(3\right)},\mu^{\left(3\right)},\sigma^{\left(3\right)},\gamma^{\left(3\right)},\beta^{\left(3\right)})\\ & +BN(M_{1}*W^{\left(1\right)},\mu^{\left(1\right)},\sigma^{\left(1\right)},\gamma^{\left(1\right)},\beta^{\left(1\right)})\\ & +BN(M_{1},\mu^{\left(0\right)},\sigma^{\left(0\right)},\gamma^{\left(0\right)},\beta^{\left(0\right)}) \end{array}$$

where $M_{1}$ is the input feature, which enters the 3 × 3 convolution branch, the 1 × 1 convolution branch, and the residual branch; ∗ denotes the convolution operation; $W^{\left(3\right)}$ is the 3 × 3 convolution kernel and $W^{\left(1\right)}$ is the 1 × 1 convolution kernel; and μ, σ, γ, and β denote the four parameters of mean, standard deviation, scale factor, and bias in the BN layer, where the superscript indicates which branch they belong to. The output feature $M_{2}$ is obtained by summing up the three branches.

### 2.4. Shuffle Attention Network

Attention mechanisms play a crucial role in enhancing the efficiency of deep neural networks, enabling them to accentuate the most informative features while suppressing the representation of less informative features. In computer vision research, there are two widely utilized attention mechanisms: spatial attention, which captures pairwise relationships at the feature pixel level, and channel attention, which concentrates on the dependencies between feature channels.

**Figure 7:**
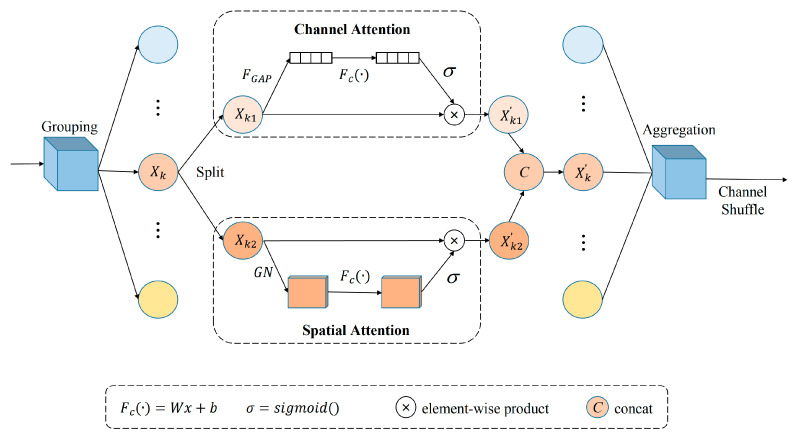
SA block structure. The SA block groups the features and divides each sub-feature equally into two groups. The two attention branches process the two groups of features and stitch the features together. The sub-features are finally reaggregated.

The architecture of the SA block is shown in Figure 7. A feature grouping is performed by the SA module for a given feature map $X\in {\mathbb{R}}^{C\times H\times W}$, where C, H, and W denote the number of channels, spatial height, and width, respectively. SA first divides *X* into *G* groups along the channel dimension—namely, $X=[X_{1},\;\cdot \cdot \cdot \;,X_{G}]$, where $X_{k}\in {\mathbb{R}}^{C/G\times H\times W}$—and each sub-feature will gradually capture a specific semantic response during training. Then, at the beginning of each attention unit, $X_{k}$ is split into two branches, $X_{k1},\;X_{k2}\in {\mathbb{R}}^{C/2G\times H\times W}$. One branch generates a channel attention map $X_{k1}^{\prime }$ by exploiting the relationships between channels, while the other branch generates a spatial attention map $X_{k2}^{\prime }$ by exploiting the spatial relationships between features. These two branches are then concatenated and, when all branches are finally aggregated, the feature information is cross-swapped in the channel dimension to obtain an output feature having the same size as the input feature.

### 2.5. Sheep Face Recognition Model

**Figure 8:**
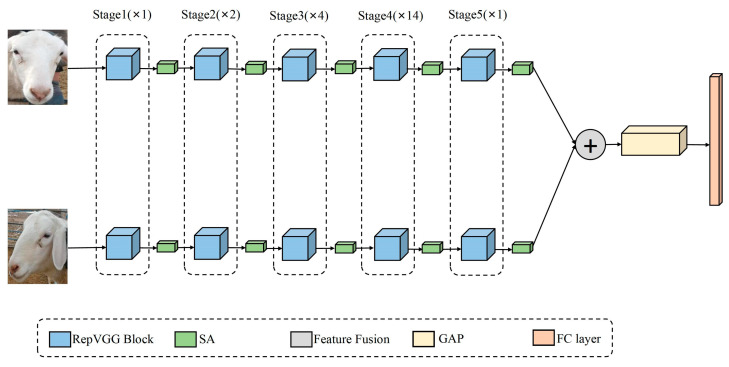
RepB-Sheepnet structure.

Figure 8 depicts our designed bilinear sheep face recognition model (RepB-Sheepnet). The model feeds a front-angle image of a sheep with a pixel size of 224 × 224 and a side-angle image of a sheep into the two feature extraction networks. The feature extraction network uses RepVGG blocks in five stages, where the number of RepVGG blocks used in each stage is 1, 2, 4, 14, and 1. The first layer of each stage downsamples the image with a stride of 2. After each stage, the SA block is embedded to learn and calibrate the feature information in order to improve the feature extraction effect. After the image passes through two feature extraction networks, it will be fused through the feature fusion layer to obtain a new feature, which is used as input for the global average pooling layer [35]. The role of the global average pooling layer is to reduce the dimensionality and regularize the structure of the entire network to prevent overfitting, as well as endowing each channel with the actual category meaning, which greatly reduces the number of parameters in the network. After the fusion features pass through the global average pooling layer, the sheep are finally classified through the fully connected layer.

### 2.6. Experimental Environment and Initial Parameters

The experimental hardware environment used in this study was an Intel Xeon Silver 4116 CPU with a main frequency of 2.1 GHz, 64 GB of memory, and an NVIDIA Corporation GP102 GPU. The software platform was the CentOS 7.9 system, with image preprocessing and network model construction and training conducted using Pytorch, implemented in Python 3.7.

The initial learning rate in the experiment was 0.0001, which was dynamically adjusted using the cosine annealing algorithm after the experiment started. The optimizer used was Adam, the number of samples per batch was set to 32 during training, and the number of training update iterations was 150.

### 2.7. Experimental Evaluation Index

Accuracy, precision, recall, and *F*1 score were calculated to evaluate the model quality. Accuracy is the percentage of correct predictions out of the total samples. Precision indicates how many of the samples predicted to be positive are truly positive samples. Recall indicates how many of the positive cases in the sample were predicted correctly. The *F*1 score is a weighted evaluation of both precision and recall.

(3) $$Accuracy=\frac{\left(TP+TN\right)}{\left(TP+TN+FP+FN\right)},$$

(4) $$Precision=\frac{TP}{\left(TP+FP\right)},$$

(5) $$Recall=\frac{TP}{\left(TP+FN\right)},$$

(6) $$F1-score=2\cdot \frac{Precision\cdot Recall}{Precision+Recall},$$

where true positive (TP) represents the number of instances that are actually positive and are classified as positive by the model, false negative (FN) refers to the number of instances that are actually positive but are classified as negative by the model, false positive (FP) represents the number of instances that are actually negative but are classified as positive by the model, and true negative (TN) refers to the number of instances that are actually negative and are classified as negative by the model.

## 3. Results

### 3.1. Sheep Face Recognition Model Training and Evaluation

**Figure 9:**
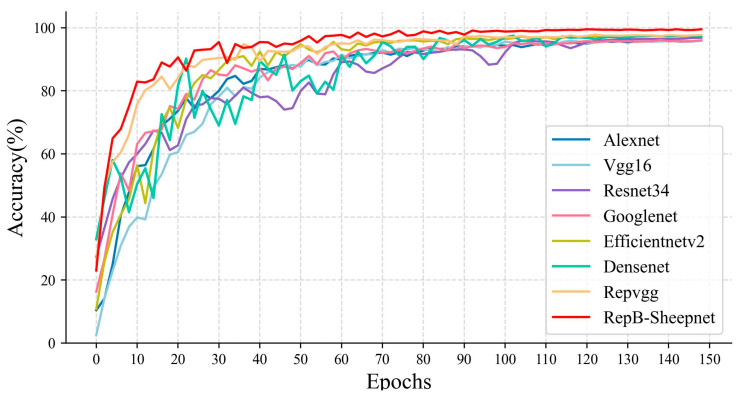
Comparison of training accuracy variation of 8 models.

**Figure 10:**
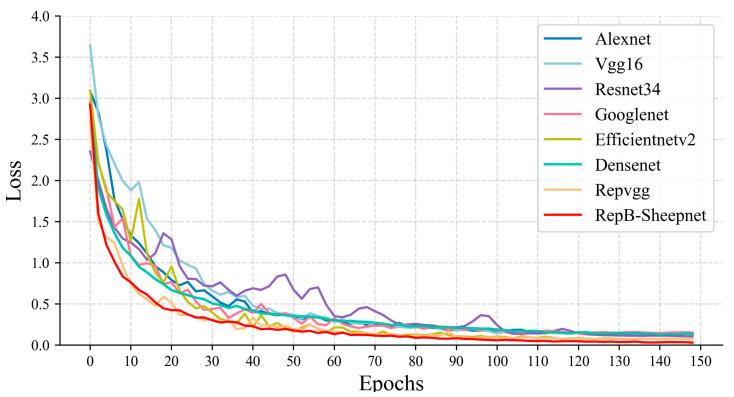
Comparison of training loss variation of 8 models.

In this study we organized and used eight models: Alexnet, VGG16, Resnet34, Googlenet, EfficientnetV2, Densenet, RepVGG, and RepB-Sheepnet. These eight models and three sheep face datasets were used for classification experiments and to compare model performance. The accuracy variation and loss variation during the model training are shown in Figure 9 and Figure 10.

Figure 9 shows the accuracy variation of the eight models during training. From the figure, it can be seen that the accuracy of RepB-Sheepnet improves the fastest and converges relatively fast. It leveled off in accuracy after 80 epochs, while the other models mainly converged gradually after 100 epochs. Figure 10 shows the training loss of each model. It is clear from the figure that RepB-Sheepnet has the smallest loss and the curve keeps decreasing smoothly, while the other models have a large oscillation. Through these two figures, it can be seen that RepB-Sheepnet has the characteristics of fast training convergence and good stability compared to the other models.

**Figure 11:**
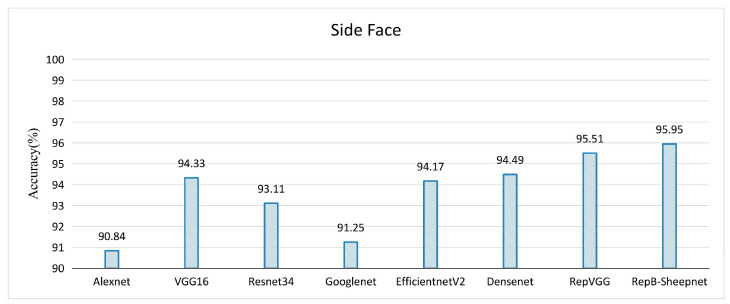
Validation accuracy of 8 recognition models on the sheep side-face dataset.

**Figure 12:**
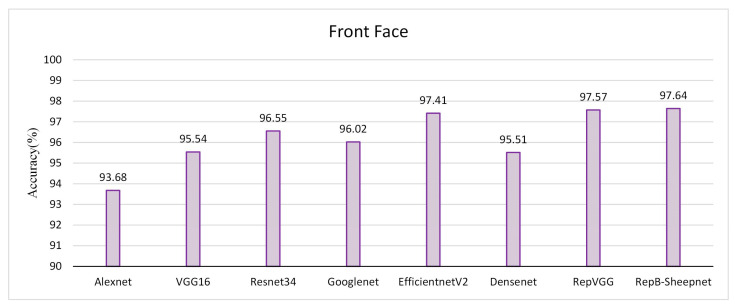
Validation accuracy of 8 recognition models on the sheep front-face dataset.

**Figure 13:**
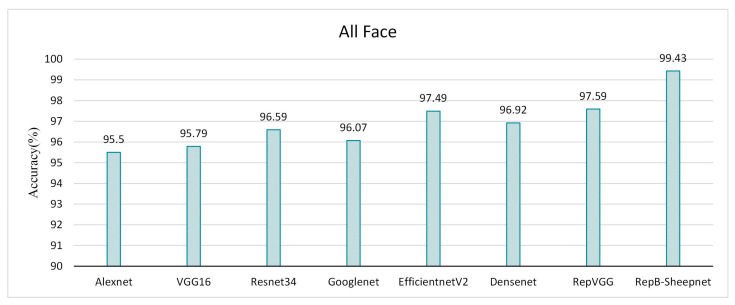
Validation accuracy of 8 recognition models on the sheep full-face dataset.

The recognition accuracy of the model was evaluated by three datasets, as shown in Figure 11, Figure 12 and Figure 13. It was found from the three figures that with the same model, the accuracy of the model tested tends to be different depending on the dataset. The model accuracy is highest using the sheep full-face dataset, and is followed by the one using the front-face dataset, while the model accuracy is worst for the side-face dataset. The accuracy of RepB-Sheepnet is the highest in all three datasets. RepB-Sheepnet achieves an accuracy of 99.43% on the sheep full-face dataset, which is the best recognition result among all the tested results.

### 3.2. Performance Testing of Different Models

**Figure 14:**
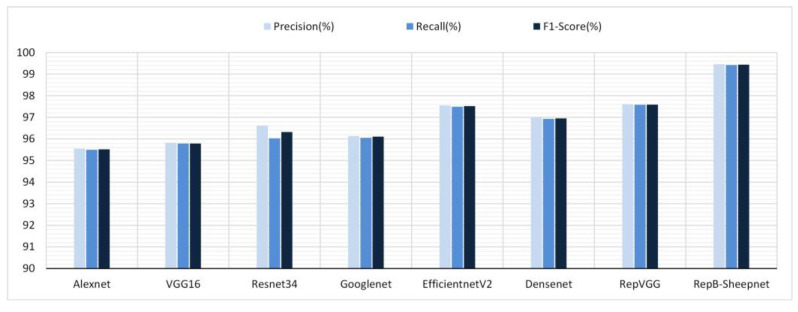
Precision, recall, and *F*1-score performance of 8 recognition models on the sheep full-face dataset.

Given the excellent performance of each model on the sheep full-face dataset, the sheep full-face dataset was selected as the dataset to use to test the performance of the models. The precision, recall and *F*1-score of the eight models tested on the dataset are shown in Figure 14. From Figure 14, it can be seen that RepB-Sheepnet has the best performance, and it has the highest precision, recall, and *F*1-score. Compared with the worst model, Alexnet, the accuracy, recall, and *F*1-score were improved by 3.91%, 3.92% and 3.92%, respectively, when using RepB-Sheepnet.

Table 1 shows the forward inference time and model parameters for each model. In terms of parameters, Googlenet has the lowest model parameters of 6.01 M, while VGG16 has the highest model parameters of 134.42 M. RepB-Sheepnet uses a two-channel structure, and thus the model parameters are 25.67 M before the combination of parameters, which is about twice as much as that of RepVGG. In terms of inference time, Alexnet is the fastest, recognizing a single image in 6.35 ms. Before structural reparameterization, RepB-Sheepnet takes 30.46 ms to recognize a single image. After structural reparameterization of the model, the recognition time (15.31 ms) is approximately halved.

### 3.3. Effect of Attention Module and Dual-Feature Extraction on Model Performance

Ablation experiments: To investigate the effectiveness of multichannel feature extraction fusion and SA attention blocks on RepB-Sheepnet, we designed four different models. Model 1: the model was built with RepVGG blocks only, without using bilinear feature extraction and without using SA blocks. Model 2: the model used SA blocks on the basis of model 1. Model 3: the model used the bilinear feature channel to extract features, but did not apply the SA block. Model 4: the model used our proposed RepB-Sheepnet model. The experimental results are shown in Table 2, from which it can be seen that the accuracy increases by 0.75% when the SA block is used in the model and by 1.43% when the bilinear feature extraction is used. In the case of using both mechanisms, the accuracy increased by 1.84%.

### 3.4. Example of Identification

**Figure 15:**
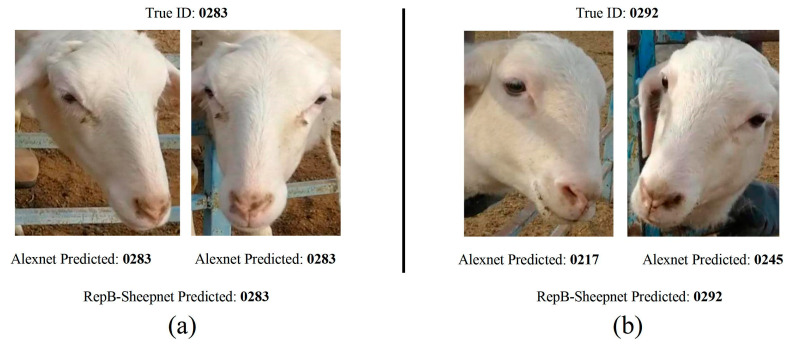
(**a**,**b**) Sheep recognition examples with two images for each sheep. The recognition models are Alexnet and RepB-Sheepnet.

Figure 15 shows the recognition results of sheep faces by Alexnet and RepB-Sheepnet. From Figure 15a, it can be seen that both Alexnet and RepB-Sheepnet recognize the sheep’s identifying information accurately. Figure 15b shows a sheep face image with a more skewed angle, and the results show that Alexnet incorrectly identifies the sheep’s identifying information. In contrast, the RepB-Sheepnet model, which uses a combination of front and side angles of sheep faces for recognition, is accurate.

## 4. Discussion

Compared with earlier sheep face recognition studies [15,26,27], this study uses multi-angle and multi-pose sheep face data, and it can be seen from Figure 11 and Figure 12 that the angle of the sheep face can affect the recognition accuracy. Based on this finding, this paper proposes a sheep face recognition model based on the RepVGG block and SA attention mechanism, which can identify individual sheep quickly and effectively under non-contact conditions. The facial features of sheep from front and side angles are extracted by two CNN channels, and then linearly fused for recognition. As seen in Figure 13 and Figure 14, the model not only reduces the influence of the sheep face pose angle on recognition, but also significantly improves the performance in recognizing sheep faces.

According to Table 1 and Table 2, compared to models using traditional CNN structures [21,22,23,25], the RepVGG blocks used in this study possess the ability to recombine parameters. The multibranch model structure is used to improve the model accuracy during training, and switching to a single-branch structure during inference cuts the time in approximately half, striking a good balance between accuracy and efficiency. The ablation experiments demonstrate that the use of SA blocks further improves the recognition accuracy of the model, which can be attributed to the fact that SA blocks can help the model improve its ability to extract global features.

As can be seen in Figure 15, the model proposed in this study has good robustness and generalization ability, makes full use of the sheep face data, and effectively reduces the effects of facial pose changes and partial feature loss in sheep face recognition. In further research, as the number of sheep to be recognized increases, the use of the ArcFace loss function in the model can be used, which performs well in the field of large-scale facial recognition [36]. The model can also use a more lightweight CNN architecture to reduce the complexity cost of the recognition model, making it easier to deploy on edge devices [37,38]. If farms want to use the model in the future, it is necessary to consider the recognition of sheep outside the dataset. In this case, incremental recognition models [39] can be considered, which can learn new features based on existing models and do not take too much time. The ability of the target detection algorithm [24,31] to remove background interference in sheep face recognition also deserves careful evaluation in the future.

## 5. Conclusions

In this paper, we collected sheep face data and created three sheep face datasets. Based on these sheep face datasets, we proposed a convolutional neural network model for sheep face recognition. The model extracts the features of different sheep faces separately via two feature extraction channels for fusion recognition, which makes full use of the sheep face data and reduces the influence of pose on the recognition results. The experimental results show that the best recognition accuracy of the model is 99.43% and the fastest time to recognize a sheep is 15.31 ms. However, the proposed method has some drawbacks, such as there being too few sheep breeds included in the dataset, and thus the accuracy rate in some complex cases will be reduced. In the future, we will continue to expand the dataset and improve the network structure in order to develop a mature sheep face recognition model.

## Figures and Tables

**Table 1:** Comparison of model inference time and parameters. The inference time of the model is the time required for the model to go from the input image to the output result, and the number of model parameters is the total number of all trainable parameters (i.e., weights and biases) in the neural network model. The inference time and parameters of RepB-Sheepnet after structural reparameterization are highlighted in bold font.

Model	Alexnet	VGG16	Resnet34	Googlenet	EfficientnetV2	Densenet	RepVGG	RepB-Sheepnet
Time (ms)	6.35	23.10	11.20	12.37	30.75	25.27	12.42	30.46/**15.31**
Parameters (M)	14.66	134.42	21.35	6.01	20.23	6.99	12.86	25.67/**23.07**

**Table 2:** Ablation experiments. “/” means that the structure is not used and “on” means that this structure is used in the network. Model numbers 1–4 represent four models, with each model structure determined according to “/” and “on”.

Model	Bilinear	SA	Accuracy (%)
1	/	/	97.59
2	/	on	98.34
3	on	/	99.02
4	on	on	99.43

## Data Availability

Not applicable.

## References

[1] Gelasakis A.I., Valergakis G.E., Arsenos G., Banos G. (2012). Description and Typology of Intensive Chios Dairy Sheep Farms in Greece. J. Dairy Sci..

[2] Morris J.E., Cronin G.M., Bush R.D., Morris J.E., Cronin G.M., Bush R.D. (2012). Improving Sheep Production and Welfare in Extensive Systems through Precision Sheep Management. Anim. Prod. Sci..

[3] Riaboff L., Aubin S., Bédère N., Couvreur S., Madouasse A., Goumand E., Chauvin A., Plantier G. (2019). Evaluation of Preprocessing Methods for the Prediction of Cattle Behaviour from Accelerometer Data. Comput. Electron. Agric..

[4] Kleen J.L., Guatteo R. (2023). Precision Livestock Farming: What Does It Contain and What Are the Perspectives?. Animals.

[5] Hatam-Nahavandi K., Carmena D., Rezaeian M., Mirjalali H., Rahimi H.M., Badri M., Vafae Eslahi A., Shahrivar F.F., Rodrigues Oliveira S.M., Pereira M.d.L. (2023). Gastrointestinal Parasites of Domestic Mammalian Hosts in Southeastern Iran. Vet. Sci..

[6] Lay D.C., Friend T.H., Bowers C.L., Grissom K.K., Jenkins O.C. (1992). A Comparative Physiological and Behavioral Study of Freeze and Hot-Iron Branding Using Dairy Cows1. J. Anim. Sci..

[7] Bai H., Zhou G., Hu Y., Sun A., Xu X., Liu X., Lu C. (2017). Traceability Technologies for Farm Animals and Their Products in China. Food Control.

[8] Reiners K., Hegger A., Hessel E.F., Böck S., Wendl G., Van den Weghe H.F.A. (2009). Application of RFID Technology Using Passive HF Transponders for the Individual Identification of Weaned Piglets at the Feed Trough. Comput. Electron. Agric..

[9] Ruiz-Garcia L.T., Lunadei L. (2011). The Role of RFID in Agriculture: Applications, Limitations and Challenges. Comput. Electron. Agric..

[10] Karakuş M., Karakuş F. (2017). The Use of Infrared Thermography for Welfare Assessment during the Application of Ear Tags to Lambs. Arch. Anim. Breed..

[11] De Luis-Garcıia R., Alberola-López C., Aghzout O., Ruiz-Alzola J. (2003). Biometric Identification Systems. Signal Process. Breed..

[12] Wildes R.P., Asmuth J.C., Green G.L., Hsu S.C., Kolczynski R.J., Matey J.R., McBride S.E. (1996). A Machine-Vision System for Iris Recognition. Mach. Vis. Appl..

[13] Choras R.S. Hybrid Iris and Retina Recognition for Biometrics. Proceedings of the 2010 3rd International Congress on Image and Signal Processing.

[14] Vasilescu M.A.O., Terzopoulos D. Multilinear Image Analysis for Facial Recognition. Proceedings of the 2002 International Conference on Pattern Recognition.

[15] Corkery G.P., UGonzales-Barron A., Butler F., Mc Donnell K., Ward S. (2007). A Preliminary Investigation on Face Recognition as a Biometric Identifier of Sheep. Trans. ASABE.

[16] Kim H.T., Ikeda Y., Choi H.L. (2005). The Identification of Japanese Black Cattle by Their Faces. Asian-Australas. J. Anim. Sci..

[17] Cai C., Li J. Cattle Face Recognition Using Local Binary Pattern Descriptor. Proceedings of the 2013 Asia-Pacific Signal and Information Processing Association Annual Summit and Conference.

[18] Kumar S., Tiwari S., Singh S.K. Face Recognition for Cattle. Proceedings of the 2015 Third International Conference on Image Information Processing (ICIIP).

[19] Wada N., Shinya M., Shiraishi M. (2013). Letter Pig Face Recognition Using Eigenspace Method. ITE Trans..

[20] Li Z., Liu F., Yang W., Peng S., Zhou J. (2021). A Survey of Convolutional Neural Networks: Analysis, Applications, and Prospects. IEEE Trans. Neural Netw. Learn. Syst..

[21] Hansen M.F., Smith M.L., Smith L.N., Salter M.G., Baxter E.M., Farish M., Grieve B. (2018). Towards On-Farm Pig Face Recognition Using Convolutional Neural Networks. Comput. Ind..

[22] Wang K., Chen C., He Y. (2020). Research on Pig Face Recognition Model Based on Keras Convolutional Neural Network. IOP Conf. Ser. Earth Environ..

[23] Marsot M., Mei J., Shan X., Ye L., Feng P., Yan X., Li C., Zhao Y. (2020). An Adaptive Pig Face Recognition Approach Using Convolutional Neural Networks. Comput. Electron. Agric..

[24] Yao L., Hu Z., Liu C., Liu H., Kuang Y., Gao Y. (2019). Cow Face Detection and Recognition Based on Automatic Feature Extraction Algorithm. Proceedings of the ACM Turing Celebration Conference—China.

[25] Wang H., Qin J., Hou Q., Gong S. (2020). Cattle Face Recognition Method Based on Parameter Transfer and Deep Learning. J. Phys. Conf. Ser..

[26] Salama A., Hassanien A.E., Fahmy A. (2019). Sheep Identification Using a Hybrid Deep Learning and Bayesian Optimization Approach. IEEE Access.

[27] Xue H., Qin J., Quan C., Ren W., Gao T., Zhao J. (2021). Open Set Sheep Face Recognition Based on Euclidean Space Metric. Math. Probl. Eng..

[28] Saradha S., Asha J., Sreemathy J. A Deep Learning-Based Framework for Sheep Identification System Based on Facial Bio-Metrics Analysis. Proceedings of the 2022 Sixth International Conference on I-SMAC (IoT in Social, Mobile, Analytics and Cloud) (I-SMAC).

[29] Yang Q.-L.Z.Y.-B. SA-Net: Shuffle Attention for Deep Convolutional Neural Networks. Proceedings of the ICASSP 2021—2021 IEEE International Conference on Acoustics, Speech and Signal Processing (ICASSP).

[30] JOCHER (2020). Network Data. https://github.com/ultralytics/yolov5.

[31] Xu X., Li W., Ran Q., Du Q., Gao L., Zhang B. (2018). Multisource Remote Sensing Data Classification Based on Convolutional Neural Network. IEEE Trans. Geosci. Remote Sens..

[32] Weng Z., Meng F., Liu S., Zhang Y., Zheng Z., Gong C. (2022). Cattle Face Recognition Based on a Two-Branch Convolutional Neural Network. Comput. Electron. Agric..

[33] Ding X., Zhang X., Ma N., Han J., Ding G., Sun J. RepVGG: Making VGG-Style ConvNets Great Again. Proceedings of the IEEE/CVF Conference on Computer Vision and Pattern Recognition.

[34] Simonyan K., Zisserman A. (2015). Very Deep Convolutional Networks for Large-Scale Image Recognition. arXiv.

[35] Lin M., Chen Q., Yan S. (2013). Network in Network. arXiv.

[36] Hitelman A., Edan Y., Godo A., Berenstein R., Lepar J., Halachmi I. (2022). Biometric Identification of Sheep via a Machine-Vision System. Comput. Electron. Agric..

[37] Pang Y., Yu W., Zhang Y., Xuan C., Wu P. (2023). Sheep Face Recognition and Classification Based on an Improved MobilenetV2 Neural Network. Int. J. Adv. Robot. Syst..

[38] Li X., Du J., Yang J., Li S. (2022). When Mobilenetv2 Meets Transformer: A Balanced Sheep Face Recognition Model. Agriculture.

[39] Belouadah E., Popescu A., Kanellos I. (2021). A Comprehensive Study of Class Incremental Learning Algorithms for Visual Tasks. Neural Netw..

